# Controlling stimulus sensitivity by tailoring nanoparticle core hydrophobicity†

**DOI:** 10.1039/d5bm00163c

**Published:** 2025-03-26

**Authors:** Xiao Zhang, Bowen Zhao, Shiwei Fu, Ronald S. Seruya, Hannah E. Fanos, Ashley A. Petrisor, Yilin Liu, Zixin Yang, Fuwu Zhang

**Affiliations:** a Department of Chemistry, University of Miami 1301 Memorial Drive Coral Gables Florida 33146 USA fxz147@miami.edu; b Sylvester Comprehensive Cancer Center, University of Miami Miller School of Medicine Miami Florida 33136 USA; c The Dr John T. Macdonald Foundation Biomedical Nanotechnology Institute, University of Miami 1951 NW 7th Ave Miami Florida 33136 USA

## Abstract

Cancer remains a significant global health challenge, necessitating the development of more effective therapeutic strategies. This work presents a novel glutathione (GSH)-responsive platform designed to enhance the delivery and efficacy of the anticancer drug mertansine (DM1) through the modulation of pendant groups in polycarbonate–drug conjugates. By systematically varying the hydrophobicity of the pendant groups, we investigated their effects on nanostructures, GSH sensitivity, colloidal stability, drug release profiles, and the *in vitro* anticancer efficacy of these polymeric nanoparticles, revealing that more hydrophobic pendant groups effectively reduce GSH accessibility for the nanoparticle cores, improve colloidal stability, and slow drug release rates. The results underscore the critical importance of polymer structures in optimizing drug delivery systems and offer valuable insights for future research on advanced nanomaterials with enhanced drug delivery for cancer therapies.

## Abstract 2

This study introduces a novel and efficient approach for constructing stimulus-responsive polycarbonates with tunable stimulus sensitivity by tailoring nanoparticle core hydrophobicity.

## Graphical abstract

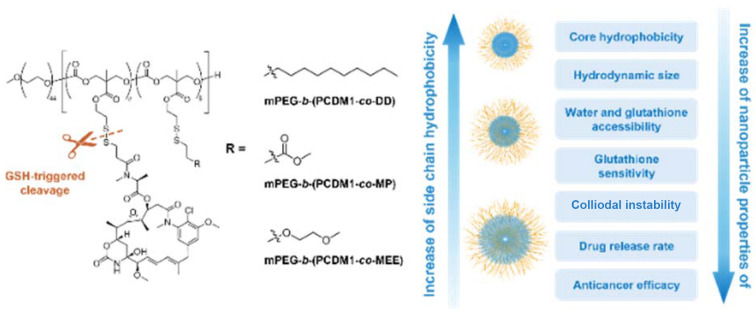

## 1. Introduction

Cancer remains a highly lethal global health challenge, and its increasing incidence necessitates the development of more effective therapeutic strategies.^1–3^ While traditional chemotherapeutic agents, particularly small molecule anticancer drugs, have demonstrated efficacy in inhibiting the proliferation and metastasis of cancer cells, their clinical applications are typically limited by severe side effects, poor solubility, and low stability under physiological conditions.^4–6^ To address these challenges, there is a pressing need for innovative delivery systems that can enhance the pharmacological properties of anticancer agents while minimizing systemic toxicity.^7^ Stimulus-responsive drug delivery systems, which are specifically engineered to respond to various internal or external stimuli such as pH, temperature, or the presence of specific biomolecules, represent a promising approach for cancer therapy.^8–14^ These systems offer the ability to maintain formulation stability during blood circulation and trigger drug release on demand, thereby significantly improving the precision of treatment and minimizing off-target effects, ultimately increasing therapeutic efficacy.^15^

Glutathione (GSH) is a naturally occurring tripeptide that plays a crucial role in various biological processes and serves as a well-studied endogenous trigger for redox-responsive drug carriers due to its distinct concentration gradients within the biological system. Intracellular GSH levels range from 2 to 10 mM, significantly exceeding extracellular concentrations of 2–20 μM, with markedly higher levels in tumor tissues compared to healthy tissues.^16–19^ Precisely controlling the sensitivity of GSH-triggered reactions is crucial for optimizing the redox-responsive drug release profile while maintaining colloidal stability during storage and *in vivo* circulation.^20–22^ Introducing steric or charged groups adjacent to the disulfide bond is an effective approach but complicates the synthetic process.^23–26^ Adjusting the position of disulfide bonds in polymeric nanomaterials and replacing them with other redox-responsive bonds, such as selenium–selenium (Se–Se) bonds, represent promising strategies; however, these approaches face challenges related to synthesis complexity and limited applicability across different platforms.^27–33^ Polymeric nanoparticles (NPs) offer significant advantages, as their colloidal stability, size, shape, flexibility, and internal accessibility can be readily adjusted by altering the chemical structure of amphiphilic polymers.^11,28^ Therefore, tuning GSH accessibility by modifying the physicochemical properties of polymeric NPs provides a straightforward approach for achieving this goal, which not only simplifies the synthesis process but also enhances adaptability in designing effective redox-responsive drug delivery systems.

**Fig. 1:**
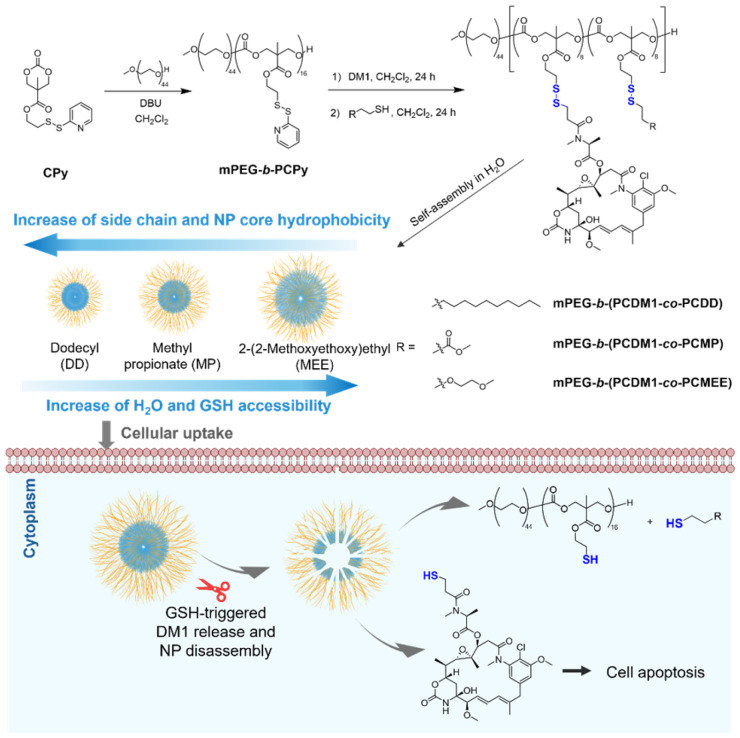
Synthetic route of the amphiphilic polycarbonate–DM1 conjugate with varying pendant groups, and their self-assembly into nanoparticles, cellular uptake and subsequent GSH-triggered release in the cell cytosol.

Aliphatic polycarbonates have drawn great attention in drug delivery due to their high biocompatibility and biodegradability. Their unique carbonate backbone (–O–C(O)–O–) can be biodegraded into small, non-toxic molecules *in vivo*, which are subsequently eliminated through natural metabolic pathways.^34–36^ Our previous research has established an efficient method for synthesizing GSH-responsive polycarbonates featuring pendant activated pyridyl disulfides, which were readily converted into redox-responsive disulfide bonds in one step with high conjugation efficiency.^37^ In this work, we utilize glutathione (GSH)-responsive polycarbonates as a platform to investigate how variations in polymer structures influence GSH accessibility for polymeric NPs and, consequently, affect the drug release profile. We covalently conjugated a highly potent anticancer drug mertansine (DM1) to the amphiphilic block copolymer methoxy poly(ethylene glycol)-*b*-polycarbonate (mPEG-*b*-PC), followed by conjugation of pendant groups with different hydrophobicities in a one-pot sequential reaction, including dodecyl (DD), methyl propionate (MP), and 2-(2-methoxyethoxy)ethyl (MEE). The differences in hydrophobicity of these pendant groups imparted varying core hydrophobicities of the self-assembled polymeric NP (Fig. 1). Incorporating more hydrophobic side chains is expected to increase the core's hydrophobicity, reducing aqueous accessibility and limiting the diffusion of water-soluble GSH, thereby hindering its ability to degrade the disulfide bonds within the polymeric matrix. This reduction in GSH sensitivity results in increased colloidal stability, slower drug release, and lower *in vitro* cytotoxicity.

## 2. Results and discussion

### 2.1 Synthesis of amphiphilic polycarbonate–DM1 conjugates

The activated cyclic carbonate monomer CPy was first synthesized according to a previously reported procedure,^37^ then subjected to ring-opening polymerization (ROP) initiated by hydrophilic methoxy poly(ethylene glycol) (mPEG-OH, *M̄*_n_ = 2000 g mol^−1^) and organocatalyzed by 1,8-diazabicyclo[5.4.0]undec-7-ene (DBU), affording the amphiphilic diblock copolymer mPEG-*b*-PCPy. The obtained polymer was purified *via* precipitation into a mixed solution of diethyl ether and hexane (1/1, v/v). The degree of polymerization (DP) of the PCPy block was determined to be 16 by ^1^H NMR spectroscopy (Fig. S1†), which is in agreement with the calculation based on a 73% monomer conversion and an initial monomer-to-initiator feeding ratio ([M]_0_/[I]_0_) of 22. One-pot postpolymerization reactions were employed to conjugate the hydrophobic anticancer drug DM1 and subsequently another pedant group of varying hydrophobicity. Briefly, the thiol-containing drug DM1 (8 equivalents relative to mPEG-*b*-PCPy) was first reacted with mPEG-*b*-PCPy in dichloromethane through a thiol–disulfide exchange reaction for 24 h. Subsequently, excess 1-dodecanethiol, methyl-3-mercaptopropionate, or 2-(2-methoxyethoxy)ethanethiol was added, and the mixture was stirred at room temperature for an additional 24 h. After evaporation of the organic solvent, the residue was redissolved in a small amount of dichloromethane (less than 5 mL) and precipitated into a mixed solution of diethyl ether and hexane (40 mL, 1/1, v/v) to remove unreacted small molecules and byproducts. After precipitating five times and drying under vacuum, the GSH-responsive polycarbonate–drug conjugates were obtained with a high yield of over 55%, which are named mPEG-*b*-(PCDM1-*co*-PCDD), mPEG-*b*-(PCDM1-*co*-PCMP), and mPEG-*b*-(PCDM1-*co*-PCMEE), respectively. The degrees of DM1 conjugation were calculated using ^1^H NMR by comparing the integrals of the methylene protons originating from the PEG segments (4.1–4.5 ppm) with the ethenyl and benzyl protons from DM1 (5.0–7.5 ppm), indicating that an average of eight DM1 molecules were conjugated to each polymer chain. The conjugation of dodecyl, methyl propionate, and 2-(2-methoxyethoxy)ethyl was confirmed by the appearance of oxygen-adjacent, carboxyl-adjacent, and methylene proton peaks in ^1^H NMR (Fig. S2–S5†) and larger dispersities by size exclusion chromatography (SEC, Fig. S6†). Additionally, the disappearance of peaks between 7.7 and 8.5 ppm confirmed the complete replacement of pyridine by the thiol-containing DM1 and pendant groups. It is worth noting that all the polycarbonate–drug conjugates demonstrated extremely high drug loading (mass of DM1/mass of the polymer–drug conjugate), achieving 45% for mPEG-*b*-(PCDM1-*co*-PCDD), 48% for mPEG-*b*-(PCDM1-*co*-PCMP), and 47% for mPEG-*b*-(PCDM1-*co*-PCMEE).

### 2.2 Self-assembly of polycarbonate–DM1 conjugates into NPs

**Fig. 2:**
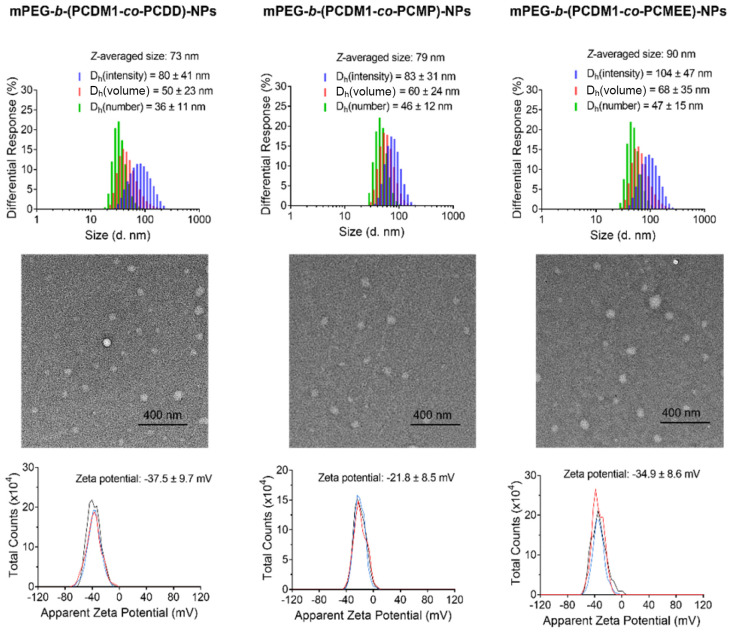
Characterization of mPEG-*b*-(PCDM1-*co*-PCDD)-NPs, mPEG-*b*-(PCDM1-*co*-PCMP)-NPs, and mPEG-*b*-(PCDM1-*co*-PCMEE)-NPs. The size distribution (top) and zeta potentials (bottom) of NPs were measured by DLS, and their morphology (middle) was imaged by TEM.

The amphiphilic nature of these polycarbonate–drug conjugates stems from their structures: the drug-linked polycarbonate block functions as the hydrophobic component, while the mPEG chain serves as the hydrophilic counterpart. The introduction of different pendant groups can provide varying degrees of hydrophobicity to the polycarbonate block, potentially endowing it with distinct physicochemical properties, especially accessibility for water soluble small molecules to their self-assembled nanoparticles. The polymer assemblies (0.1 mg mL^−1^) were prepared by nanoprecipitation from THF into pure water, followed by evaporation of organic solvent and filtration to remove undissolved aggregates. The *Z*-averaged diameters of these nanoparticles, measured by dynamic light scattering (DLS), ranged from 73 to 90 nm, which are optimal sizes for evading rapid clearance during blood circulation while ensuring good cellular uptake (Fig. 2).^38,39^ The spherical structures of NPs were confirmed by transmission electron microscopy (TEM). Moreover, an increase in NP sizes was observed with decreasing hydrophobicity of the pendant groups. Specifically, mPEG-*b*-(PCDM1-*co*-PCDD)-NPs with most hydrophobic dodecyl groups exhibited the smallest hydrodynamic diameter of 73 nm, while mPEG-*b*-(PCDM1-*co*-PCMEE)-NPs bearing the least hydrophobic 2-(2-methoxyethoxy)ethyl groups showed the largest diameter of 90 nm. The mPEG-*b*-(PCDM1-*co*-PCMP)-NPs, featuring a medium hydrophobic methyl propionate, had a hydrodynamic diameter of 79 nm. These variations reflect that the core of mPEG-*b*-(PCDM1-*co*-PCDD)-NPs may be the most compact, likely due to the higher hydrophobicity of the dodecyl groups compared to the methyl propionate and 2-(2-methoxyethoxy)ethyl groups. Additionally, all the nanoparticles exhibited negative zeta potentials at around −30 mV (Fig. 2), which helps stabilize the nanoparticles by creating electrostatic repulsion to prevent undesirable aggregation in aqueous solutions.^40,41^

### 2.3 Water accessibility for the core of self-assembled nanoparticles

**Fig. 3:**
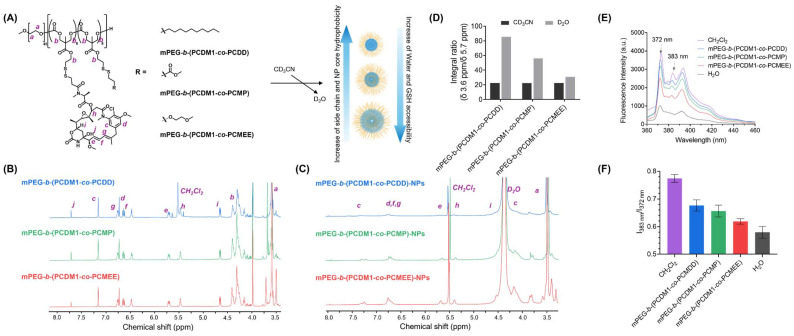
(A) Schematic representation of the preparation of self-assembled NPs for evaluating water and GSH accessibility using ^1^H NMR spectroscopy. (B) ^1^H NMR spectra of the synthesized polymers: mPEG-*b*-(PCDM1-*co*-PCDD) (blue), mPEG-*b*-(PCDM1-*co*-PCMP) (green), and mPEG-*b*-(PCDM1-*co*-PCMEE) (red) in CD_3_CN. (C) ^1^H NMR spectra of the self-assembled nanoparticles: mPEG-*b*-(PCDM1-*co*-PCDD)-NPs (blue), mPEG-*b*-(PCDM1-*co*-PCMP)-NPs (green), and mPEG-*b*-(PCDM1-*co*-PCMEE)-NPs (red) in D_2_O (containing 30% CD_3_CN). (D) Integral ratios (*δ* 3.6 ppm/*δ* 5.7 ppm) for polymers in CD_3_CN (black bar) and nanoparticles in D_2_O (grey bar), with peaks at 3.6 ppm representing methylene protons of PEG segments and peaks at 5.7 ppm representing ethenyl protons of DM1. (E) Fluorescence spectrum of pyrene (0.12 μg mL^−1^) in various aqueous solutions of different polymers (0.1 mg mL^−1^), in the hydrophobic solvent CH_2_Cl_2_, and in pure H_2_O. (F) Fluorescence intensity ratios (*I*_383 nm_/*I*_372 nm_) of pyrene in the above solutions, illustrating the different microenvironmental polarity of NP cores.

The microenvironment of disulfide bonds significantly impacts the kinetics of the GSH–disulfide exchange reaction.^26,42^ In our design, conjugating different pendant groups to adjust GSH accessibility for the hydrophobic core of nanoparticles, where the redox-responsive disulfide bonds are located, is crucial for optimizing the stability and stimulus-responsiveness of drug delivery systems. Optimal GSH accessibility will help maintain the stability of the nanoparticles, preventing premature drug release during systemic circulation and ensuring triggered release at targeted therapeutic sites. To investigate GSH accessibility for the hydrophobic cores of our prepared nanoparticles, we evaluated the local microenvironment of disulfide bonds using the solution-state ^1^H NMR spectroscopy, which provided an insight into the aqueous accessibility of the hydrophobic polycarbonate segments when packaged within nanostructures in D_2_O *vs.* as solvated polymer chains in CD_3_CN (Fig. 3A–D). The differences can be quantified by the ratio of the ^1^H NMR integrations between PEG-associated methylene protons (H^a^) resonating at 3.6 ppm and DM1-associated ethenyl protons (H^e^) resonating at 5.7 ppm. In pure CD_3_CN, all the polymers remained at a constant integral ratio (*δ* 3.6 ppm/*δ* 5.7 ppm) at about 22, where they were in a well-dissolved state (Fig. 3B). However, when these amphiphilic polymers self-assembled into nanoparticles in D_2_O, the ^1^H NMR peaks for their hydrophobic polycarbonate segments significantly decreased and broadened due to the reduced water accessibility and restricted mobility in the hydrophobic nanoparticle cores (Fig. 3C). The proton resonances for hydrophilic PEG segments remained unaffected since they are mainly distributed on the surface of nanoparticles, resulting in an increased integral ratio in NPs (Fig. 3C). As the hydrophobicity of the pendant groups increased from MEE to DD, the integral ratio exhibited a 2.8-fold increase from 31 to 86 in D_2_O, both higher than the ratio of 22.5 observed in pure CD_3_CN (Fig. 3D). This phenomenon suggested that more hydrophobic pendent groups contributed to greater hydrophobicity within the nanoparticle cores, leading to lower water accessibility, which likely leads to slower kinetics of the GSH–disulfide exchange reaction, and ultimately slower the GSH-triggered drug release.

The influence of pendant groups on the core hydrophobicity of the nanoparticles was further evaluated by co-incubating aqueous NP solutions with pyrene, a hydrophobic small-molecule fluorescent probe that preferably accumulated in the hydrophobic cores of NPs (Fig. 3E and F). The microenvironmental polarity of the NP cores was then evaluated by analysing the ratio of pyrene's third (383 nm) to first (372 nm) vibronic band fluorescence intensities.^43,44^ A consistent concentration of polycarbonate–DM1 conjugate nanoparticles (0.1 mg mL^−1^) was mixed with pyrene (0.12 μg mL^−1^) for 12 hours to reach equilibrium, after which the fluorescence spectra were recorded (Fig. 3E). The fluorescence intensity ratio (*I*_383 nm_/*I*_372 nm_) demonstrated a gradual decrease from mPEG-*b*-(PCDM1-*co*-PCDD) to mPEG-*b*-(PCDM1-*co*-PCMP), and finally to mPEG-*b*-(PCDM1-*co*-PCMEE) (Fig. 3F), all of which were significantly higher than the ratio observed for pyrene in pure water. This trend suggests that polycarbonate–DM1 conjugates with progressively more hydrophobic pendant chains result in increased hydrophobicity within the nanoparticle core, aligning with the results obtained from our previous ^1^H NMR analysis.

### 2.4 Colloidal stability of self-assembled nanoparticles

**Fig. 4:**
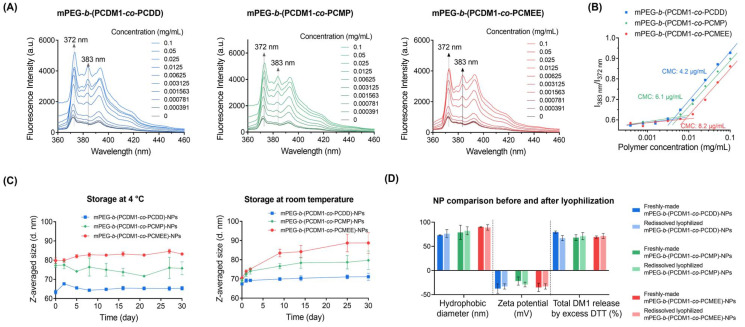
(A) Fluorescence spectra of pyrene (0.12 μg mL^−1^) in the aqueous solutions of different concentrations of polycarbonate–DM1 conjugates (0–0.1 mg mL^−1^): mPEG-*b*-(PCDM1-*co*-PCDD) (blue); mPEG-*b*-(PCDM1-*co*-PCMP) (green); and mPEG-*b*-(PCDM1-*co*-PCMEE) (red). (B) Fluorescence intensity ratio (*I*_383_ nm/*I*_372_ nm) of pyrene *versus* the logarithm concentration of polymers, with CMC determined by fitting the intensity ratio to identify the abrupt change point. (C) Long-term stability of self-assembled NPs at room temperature and under refrigerated conditions, evaluated by their *Z*-averaged size changes in DLS. (D) Comparison of sizes, zeta potentials, and DM1 release capabilities between the freshly made nanoparticles and reconstituted nanoparticles from lyophilized powders.

The stability of these self-assembled nanoparticles upon dilution was evaluated by determining the critical micelle concentration (CMC) of the amphiphilic polymers, which represents the threshold concentration above which the polycarbonate–DM1 conjugates can self-assemble into nanoparticles in an aqueous environment.^45,46^ Amphiphilic polymers with low CMC risk disassemble when diluted below their CMC, whether during sample preparation or after *in vivo* administration. To measure the CMC, pyrene was co-incubated with varying concentrations of the polymers (Fig. 4A). The polymer concentration at which a significant change in the fluorescence intensity ratio (*I*_383 nm_/*I*_372 nm_) was observed was recorded as the CMC of the polymer. It was found that mPEG-*b*-(PCDM1-*co*-PCDD) with the most hydrophobic pendant chains exhibited the lowest CMC at 4.2 μg mL^−1^, followed by mPEG-*b*-(PCDM1-*co*-PCDD) at 6.1 μg mL^−1^ and mPEG-*b*-(PCDM1-*co*-PCMEE) at 8.2 μg mL^−1^ (Fig. 4B). This suggested that mPEG-*b*-(PCDM1-*co*-PCDD) had higher colloidal stability upon dilution, which is consistent with the observation that more hydrophobic polymers typically exhibit lower CMC values and improved stability upon dilution.

Time-dependent DLS was conducted to monitor the long-term colloidal stability of polymeric nanoparticles under storage conditions at 4 °C and room temperature (Fig. 4C). All nanoparticles exhibited exceptional colloidal stability under refrigerated conditions, remaining largely unchanged for at least 30 days. At room temperature over 30 days, the hydrodynamic size of mPEG-*b*-(PCDM1-*co*-PCDD)-NPs remained stable. In contrast, both mPEG-*b*-(PCDM1-*co*-PCMEE)-NPs and mPEG-*b*-(PCDM1-*co*-PCMP)-NPs exhibited increased hydrodynamic diameters and dispersities, indicative of gradual aggregation during storage. The stability trend was measured as follows: mPEG-*b*-(PCDM1-*co*-PCDD)-NP > mPEG-*b*-(PCDM1-*co*-PCMP)-NP > mPEG-*b*-(PCDM1-*co*-PCMEE)-NP. This order of stability correlates well with the hydrophobicity of pendant groups conjugated to the polycarbonate backbones, suggesting that nanoparticles with more hydrophobic pendant groups are better at maintaining their nanostructure and resisting aggregation over time. Additionally, all nanoparticles self-assembled from these polycarbonate–drug conjugates could be lyophilized into a dry state for storage, with no significant changes observed upon reconstitution in aqueous solutions (Fig. 4D). This lyophilization capability is a highly desirable feature for nanoparticle (NP) formulations, as it ensures stability and facilitates long-term storage.

### 2.5 GSH-triggered drug release from self-assembled NPs and their anticancer efficacies

**Fig. 5:**
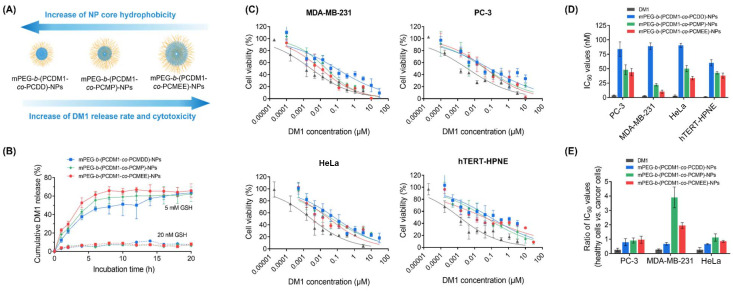
(A) Relationship between the polymer side chain hydrophobicity, drug release rate, and *in vitro* cytotoxicity towards cancer cells. (B) The release profile of DM1 from PBS solution of mPEG-*b*-(PCDM1-*co*-PCDD)-NPs, mPEG-*b*-(PCDM1-*co*-PCMP)-NPs, and mPEG-*b*-(PCDM1-*co*-PCMEE)-NPs (20 μg mL^−1^) containing different GSH concentrations (20 nM and 5 mM). (C) *In vitro* cytotoxicity of DM1, mPEG-*b*-(PCDM1-*co*-PCDD)-NPs, mPEG-*b*-(PCDM1-*co*-PCMP)-NPs, and mPEG-*b*-(PCDM1-*co*-PCMEE)-NPs towards cancerous cell lines (MDA-MB-231, PC-3, and HeLa) and a healthy cell line (hTERT-HPNE) after 72 h of incubation at 37 °C and 5% CO_2_. (D) Calculated IC_50_ values for the tested formulations. (E) Ratios of IC_50_ values against the healthy hTERT-HPNE cell line *vs.* different cancerous cell lines.

The kinetics of GSH-triggered DM1 release were investigated in phosphate-buffered saline (PBS, pH 7.4) with different GSH concentrations (5 mM and 20 nM), and the released DM1 was quantified using high-performance liquid chromatography (HPLC) (Fig. 5A and B). The time-dependent HPLC traces indicated a relatively rapid drug release from all these polycarboante–DM1 conjugate assemblies at a GSH concentration of 5 mM, achieving nearly complete drug release within 8 h. Notably, mPEG-*b*-(PCDM1-*co*-PCMEE)-NPs with the most hydrophilic core demonstrated the fastest drug release during the first 8 h, likely due to greater water accessibility for the core resulting in higher GSH reactivity (Fig. 5B). In contrast, mPEG-*b*-(PCDM1-*co*-PCDD)-NPs with the most hydrophobic core exhibited the slowest release rate but still could achieve 50% release within 10 hours. The release behaviors in low GSH environments (20 nM), which mimic the much lower GSH concentration during blood circulation, were also examined to assess their stability during systemic administration. All assemblies remained stable for at least 20 hours without significant DM1 release (<10%). Therefore, these NP assemblies are expected to remain stable during blood circulation while allowing for rapid DM1 release in the tumors, minimizing systemic side effects and enhancing therapeutic efficacy.

**Fig. 6:**
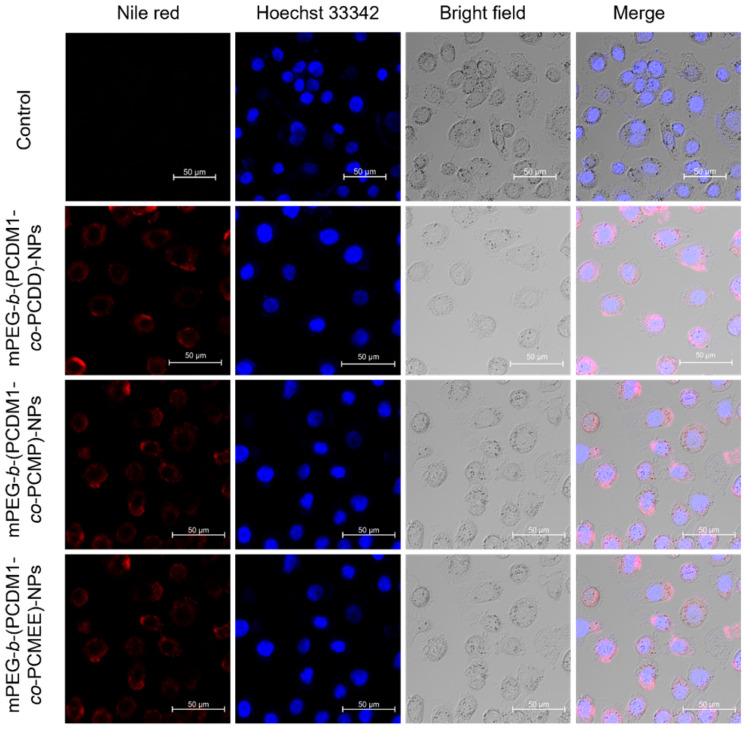
The uptake of Nile red-encapsulated mPEG-*b*-(PCDM1-*co*-PCDD)-NPs, mPEG-*b*-(PCDM1-*co*-PCMP)-NPs, and mPEG-*b*-(PCDM1-*co*-PCMEE)-NPs in PC-3 cells after incubation at 37 °C and 5% CO_2_ for 1.5 h. The Nile red encapsulated micelles were detected in the red channel, and the nuclei were stained with Hoechst 33 342 and detected in the blue channel.

The cellular uptake of these polymeric NPs was further investigated by confocal laser scanning microscopy (Fig. 6). Hydrophobic fluorophore Nile Red was co-assembled with the amphiphilic polycarbonate–DM1 conjugates in aqueous solutions and encapsulated in the hydrophobic core of the nanoparticle. The bright red fluorescence from Nile Red upon excitation allowed for direct visualization of nanoparticle behaviors in the cells.^47^ After incubating NPs with PC-3 prostate cancer cells for 1.5 hours at 37 °C in the presence of 5% CO_2_, a strong red fluorescence was well distributed in the cytoplasm, suggesting that the nanoparticles could be easily internalized by cancer cells. No significant differences were observed among these NPs. Once taken up by cancer cells, the NPs can release DM1 intracellularly, where it binds to tubulin, disrupting microtubule dynamics and ultimately inducing cell apoptosis.^37,48^

Finally, the anticancer efficacy of the polycarbonate–DM1 conjugate NP assemblies was evaluated by measuring their cytotoxicity against different cancerous cell lines (HeLa, a human cervical cancer cell line; MDA-MB-231, a human breast cancer cell line; and PC-3, a human prostate cancer cell line) (Fig. 5C and D). A healthy cell line (hTERT-HPNE, an hTERT-immortalized pancreatic epithelial cell line) was also used as a control. All cell lines were purchased from the American Type Culture Collection (ATCC). The 3-(4,5-dimethylthiazol-2-yl)-2,5-diphenyltetrazolium bromide (MTT) assay revealed that DM1 exhibited extreme cytotoxicity towards all cell lines, with IC_50_ values ranging from 0.5 to 5 nM. After conjugation to polycarbonate and self-assembly into NPs, their cytotoxicity significantly decreased due to the additional drug release process, resulting in increased IC_50_ values ranging from 24 nM to 73 nM (according to equivalent DM1 concentrations). In particular, mPEG-*b*-(PCDM1-*co*-PCDD)-NPs exhibited the most significant decrease in cytotoxicity due to its slowest drug release rate. Furthermore, all nanoparticles demonstrated considerably reduced cytotoxicity towards healthy hTERT-HPNE cells, with over 25-fold increased IC_50_ values compared to that of free DM1, indicating their improved differential cytotoxicities against cancer cells over healthy cells. To further quantify this differential cytotoxicity, we calculated the ratio of IC_50_ values for healthy hTERT-HPNE cell line *vs.* cancerous cells, where a greater ratio typically indicates a broader therapeutic window for the therapeutic agent.^16,37^ All nanoparticles exhibited obviously increased ratios compared to DM1 (Fig. 5E), demonstrating their enhanced differential cytotoxicities against cancer cells, underscoring their potential for effective cancer therapy.

## 3. Conclusions

In summary, we demonstrated the critical roles of pendant groups in modulating the hydrophobicity of the nanoparticle core and thereby tuning the micellar stability and stimulus sensitivity. Our study established a modular redox-responsive polycarbonate–drug conjugate platform through a facile and highly efficient synthetic method. By deliberately tailoring the pendant groups, we successfully tuned the core hydrophobicity of the nanoparticles, which in turn influenced their colloidal stability, drug release profiles, and ultimately their therapeutic efficacy. The results indicate that more hydrophobic pendant groups yielded nanoparticles with greater core hydrophobicity, which effectively decreased GSH accessibility for the nanoparticle core, thereby improving colloidal stability with better controlled drug release rates. By building on this strategy and framework, polymeric nanoparticles with tuneable release kinetics can be designed using straightforward methods, paving the way for developing nanomaterials with enhanced therapeutic efficacy in cancer treatment.

## Author contributions

The manuscript was written through the contributions of all authors. All authors have given approval to the final version of the manuscript.

## Data availability

The data supporting this article have been included as part of the ESI.†

## Conflicts of interest

There are no conflicts to declare.

## Supplementary Material

BM-013-D5BM00163C-s001
