# Supplementary material for: Controlling stimulus sensitivity by tailoring nanoparticle core hydrophobicity
Source: Biomater Sci. 2025 Mar 26;13(9):2332–9. doi: 10.1039/d5bm00163c (PMC11939950; doi:10.1039/d5bm00163c)
Supplement: BM-013-D5BM00163C-s001 [file BM-013-D5BM00163C-s001.pdf]

Supplementary Information (SI) for Biomaterials Science.
This journal is © The Royal Society of Chemistry 2025

Supporting Information

Controlling stimuli sensitivity through tailoring nanoparticle core hydrophobicity

Xiao Zhang,[a] *Bowen Zhao,*[a] *Shiwei Fu,* [a] *Ronald S. Seruya,* [a] *Hannah E. Fanos,* [a] *Ashley A. Petrisor,* [a] *Yilin Liu,* [a] *Zixin Yang,* [a] *and Fuwu Zhang**[abc]

[a] Department of Chemistry, University of Miami, 1301 Memorial Drive, Coral Gables, Florida, 33146, United States.

[b] Sylvester Comprehensive Cancer Center, University of Miami Miller School of Medicine, Miami, Florida 33136, United States.

[c] The Dr. John T. Macdonald Foundation Biomedical Nanotechnology Institute, University of Miami, Miami, FL 33136, United States

Corresponding Author: Dr. Fuwu Zhang, fxz174@miami.edu

Table of Contents

1. Experiment Section 1

1.1 Materials 1

1.2 Instruments 1

1.3 Synthesis of amphiphilic diblock copolymer mPEG-*b*-PCPy 2

1.4 Synthesis of polycarbonate-drug conjugates mPEG-*b*-(PCDM1-*co*-PCDD), mPEG-*b*-(PCDM1-*co*-PCMP), and mPEG-*b*-(PCDM1-*co*-PCMEE) 2

1.5 Self-assembly of amphiphilic polycarbonate-drug conjugates into nanoparticles 4

1.6 Water accessibility of self-assembled nanoparticle evaluated by ^{1}H NMR 4

1.7 Determination of critical micelle concentrations (CMCs) for mPEG-*b*-(PCDM1-*co*-PCDD), mPEG-*b*-(PCDM1-*co*-PCMP), and mPEG-*b*-(PCDM1-*co*-PCMEE) 4

1.8 Long-term stability of mPEG-*b*-(PCDM1-*co*-PCDD)-NPs, mPEG-*b*-(PCDM1-*co*-PCMP)-NPs, and mPEG-*b*-(PCDM1-*co*-PCMEE)-NPs 5

1.9 DM1 release from mPEG-*b*-(PCDM1-*co*-PCDD)-NPs, mPEG-*b*-(PCDM1-*co*-PCMP)-NPs, and mPEG-*b*-(PCDM1-*co*-PCMEE)-NPs 5

1.10 Cell cultures 6

1.11 Preparation of Nile red encapsulated polycarbonate-DM1 NPs 6

1.12 Uptake of polycarbonate-DM1 NPs 7

1.13 Evaluation of cytotoxicity of nanoparticles with MTT assay 7

2. Supplemental Figures and Tables 8

1. Experimental Section

1.1 Materials:

DM1 was purchased from Ambeed. 1,8-diazabicyclo[5.4.0]undec-7-ene (DBU) and 1,8-Octanedithiol were purchased from Sigma-Aldrich. Polyethylene glycol monomethyl ether 2000 and Nile red were purchased from TCI. Other chemicals and solvents without special instructions were purchased from VWR and used as received without further purification. All cell lines were purchased from American Type Culture Collection (ATCC). Minimum Essential Medium (MEM) with Earle's salt and L-glutamine, Dulbecco's Modification of Eagle's Medium (DMEM) with 4.5 g/L glucose, L-glutamine and sodium pyruvate, 0.25% trypsin with 2.21 mM EDTA, and Dulbecco's Phosphate Buffered Silane (DPBS) and fetal bovine serum (FBS) were purchased from Corning. Penicillin-Streptomycin solution was purchased from Cytiva. Thiazolyl blue tetrazolium bromide (MTT) was purchased from Acros Organics. Dimethyl Sulfoxide (DMSO) was purchased from Fisher Chemical. Hoechst 33342 was purchased from BD Pharmingen. Propidium Iodide (PI) was purchased from Biotium.

1.2 Instruments:

Water was purified by a Thermo Scientific Barnstead MicroPure Water Purification System containing a 0.2 μm point of dispensing filter. Proton nuclear magnetic resonance (^{1}H NMR) spectra were recorded on a 500 MHz Bruker NMR spectrometer with multinuclear inverse and broadband 5 mm probes. Gel permeation chromatography (GPC) analysis was carried out using Shimadzu GPC system containing DGU-403 degassing unit, LC-40D solvent delivery module, RID-20A refractive index detector, and CTO-40C column oven. Dynamic light scattering (DLS) measurements for nanoparticle diameter and zeta potential were carried out using a Malvern Zetasizer Nano ZS system. pH value was measured with Accumet Model 50 pH/Ion/Conductivity Meter from Fisher Scientific. High performance liquid chromatography (HPLC) analyses were made using a Shimadzu HPLC system containing DGU-405 degassing unit, LC-20AR liquid chromatography, SIL-40C auto sampler, CBM-40 system controller, CTO-40C column oven, and SPD-40 UV-vis detector. Plate reader analysis was performed on a SpectraMax i3x microplate reader from Molecular Devices. Cells were counted using a Luna-II automated cell counter (Aligned Genetics, Inc.).

1.3 Synthesis of amphiphilic diblock copolymer mPEG-b-PCPy

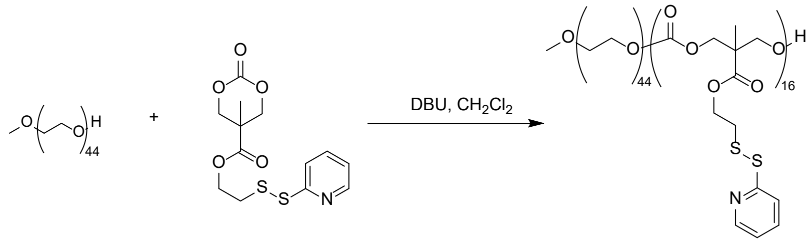

mPEG-OH **CPy** **mPEG-*b*-PCPy**

The monomer CPy was synthesized following established procedures. Subsequently, the ring-opening polymerization of CPy was initiated by poly(ethylene glycol) monomethyl ether (average Mn ~2000, mPEG-OH) and catalyzed with 1,8-Diazabicyclo[5.4.0]undec-7-ene (DBU). Specifically, mPEG-OH (30 mg, 0.015 mmol, 1 eq.) and DBU were dissolved in anhydrous CH_2Cl_2 (2 mL). The monomer CPy (108 mg, 0.33 mmol, 22 eq.) was then dissolved in 1 mL of anhydrous CH_2Cl_2 and added dropwise to the solution. After stirring at room temperature for 6 hours, the polymerization was quenched with excessive glacial acetic acid. After the evaporation of the solvent, the residue was redissolved in 2 mL of CH_2Cl_2, and 1 mL of ethanol was added. The polymer was purified through precipitation into a mixed solution of diethyl ether and hexane (40 mL, 1/1, v/v). This precipitation step was repeated five times, and the compound was dried under vacuum to yield the polymer as a colorless oil (77 mg, 56% yield). According to ^{1}H NMR analysis, the degree of polymerization was calculated to be 16, with a conversion rate of 73%. ^{1}H NMR (500 MHz, $CDCl_3$) δ: 8.46 (m, 16H), 7.73 – 7.59 (m, 32H), 7.10 (m, 16H), 4.47 – 4.20 (m, 102H), 3.64 (s, 180H), 3.03 (m, 32H), 1.33 – 1.18 (m, 48H).

1.4 Synthesis of polycarbonate-drug conjugates mPEG-b-(PCDM1-co-PCDD), mPEG-b-(PCDM1-co-PCMP), and mPEG-b-(PCDM1-co-PCMEE)

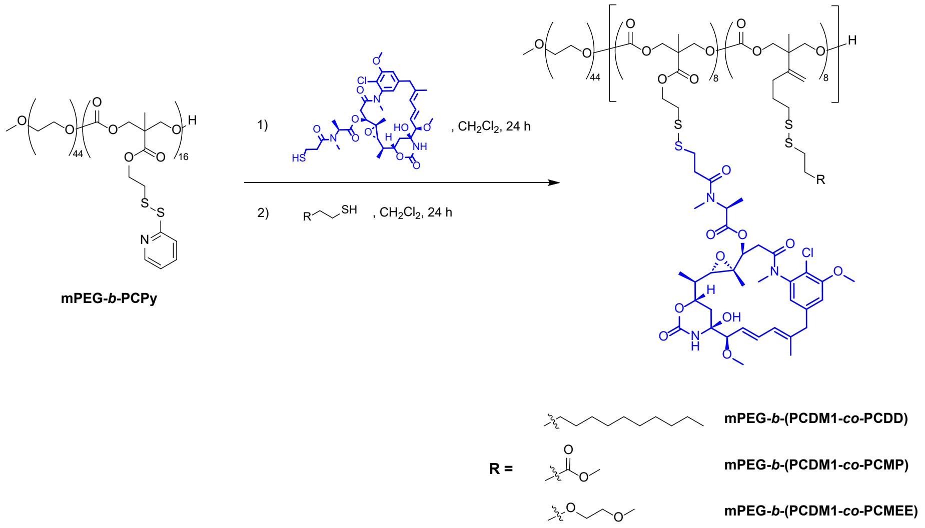

The synthesis of polycarbonate-drug conjugates mPEG-*b*-(PCDM1-*co*-PCDD), mPEG-*b*-(PCDM1-*co*-PCMP), and mPEG-*b*-(PCDM1-*co*-PCMEE) was carried out using a one-pot reaction. mPEG-*b*-PCPy (30 mg, 0.004 mmol, 1 eq.) was dissolved in 8 mL of CH_2Cl_2 in a glass vial, followed by the dropwise addition of a DM1 solution (24 mg, 0.033 mmol, 8 eq.) in CH_2Cl_2 (1 mL). The reaction was stirred at room temperature for 24 hours to ensure complete conjugation of DM1. The solution was then divided evenly into three separate vials, each containing 3 mL solution, for the addition of different pendant groups.

1-Dodecanethiol (11 mg, 0.055 mmol, 40 eq.), methyl-3-mercaptopropionate (6.6 mg, 0.055 mmol, 40 eq.), and 2-(2-methoxyethoxy)ethanethiol (7.5 mg, 0.055 mmol, 40 eq.) were dissolved in 1mL of CH_2Cl_2 separately, and then added dropwise into the respective vials. After reacting at room temperature for another 24 hours, the polycarbonate-drug conjugates were purified by precipitation from CH_2Cl_2 into a mixed solution of diethyl ether and hexane (1:1 v/v) five times to remove small molecular byproducts. After drying under vacuum, the target product was obtained as white powders or gels.

mPEG-*b*-(PCDM1-*co*-PCDD): 9.8 mg, yield 55%. ^{1}H NMR (500 MHz, $CDCl_3$) δ: 6.85 – 6.80 (m, 8H), 6.63 (br, 8H), 6.42 (d, 8H), 6.31 – 6.21 (br, 8H), 5.65 (m, 8H), 4.78 (d, *J* = 12.3 Hz, 8H), 4.45 – 4.18 (m, 96H), 3.64 (s, 180H), 3.54 – 3.44 (m, 19H), 3.36 (d, 30H), 3.28 – 3.17 (m, 32H), 3.12 (t, *J* = 13.9 Hz, 15H), 2.92 – 2.84 (m, 56H), 2.83 – 2.75 (m, 25H), 2.69 (m, 30H), 2.65 – 2.52 (m, 22H), 2.18 (dd, *J* = 14.3, 14H), 1.71 – 1.59 (m, 24H), 1.39 – 1.13 (m, 275H), 0.92 – 0.78 (m, 69H).

mPEG-*b*-(PCDM1-*co*-PCMP): 11.2 mg, yield 65%. ^{1}H NMR (500 MHz, $CDCl_3$) δ: 6.80 – 6.72 (m, 8H), 6.67 – 6.59 (br, 8H), 6.57 (m, 8H), 6.40 – 6.31 (m, 8H), 6.16 (m, 8H), 5.59 (m, 8H), 5.39 – 5.27 (m, 9H), 4.71 (d, *J* = 12.1, 8H), 4.48 – 4.03 (m, 95H), 3.92 (s, 22H), 3.64 (s, 24H), 3.57 (s, 135H), 3.45 – 3.40 (m, 9H), 3.36 – 3.25 (m, 27H), 3.16 (d, *J* = 6.8 Hz, 21H), 3.04 (d, *J* = 12.6 Hz, 9H), 2.99 – 2.63 (m, 118H), 2.63 – 2.48 (m, 18H), 2.11 (d, *J* = 14.5, 8H), 1.31 – 1.11 (m, 98H), 0.92 – 0.78 (m, 24H).

mPEG-*b*-(PCDM1-*co*-PCMEE): 10.3 mg, 59%. ^{1}H NMR (500 MHz, $CDCl_3$) δ: 6.77 (m, 8H), 6.67 – 6.59 (br, 8H), 6.57 (m, 8H), 6.36 (d, *J* = 15.3, 8H), 6.17 (br, 8H), 5.59 (m, 8H), 5.33 (m, 8H), 4.75 – 4.67 (m, 8H), 4.48 – 4.04 (m, 115H), 3.92 (s, 26H), 3.67 (m, 30H), 3.58 (s, 180H), 3.48 (t, *J* = 4.6 Hz, 24H), 3.45 – 3.40 (m, 11H), 3.37 – 3.25 (m, 60H), 3.16 (m, 25H), 3.05 (d,

8H), 2.99 – 2.65 (m, 127H), 2.65 – 2.48 (m, 16H), 2.11 (d, $J = 14.4$, 12H), 1.58 (s, 24H), 1.31 – 1.08 (m, 96H), 0.73 (s, 24H).

1.5 Self-assembly of amphiphilic polycarbonate-drug conjugates into nanoparticles

The self-assembly of amphiphilic polycarbonate-drug conjugates was conducted by dissolving 2 mg of mPEG-*b*-(PCDM1-*co*-PCDD), mPEG-*b*-(PCDM1-*co*-PCMP), and mPEG-*b*-(PCDM1-*co*-PCMEE) in 1 mL of THF, respectively, to create stock solutions of these polycarbonate-drug conjugates with a concentration of 2 mg/mL. 200 µL of the polymer stock solutions were then added dropwise into 2 mL of pure water while stirring at room temperature. After continuously stirring for 20 minutes, THF was evaporated under a nitrogen stream, and the volume was adjusted to 2 mL with pure water. The resulting aqueous solutions of mPEG-*b*-(PCDM1-*co*-PCDD), mPEG-*b*-(PCDM1-*co*-PCMP), and mPEG-*b*-(PCDM1-*co*-PCMEE) were obtained, each with a concentration of 200 µg/mL. The diameters and zeta potentials of the nanoparticles were measured by dynamic light scattering (DLS) without further purification. TEM was measured after stained with 1% aqueous uranyl acetate.

1.6 Water accessibility of self-assembled nanoparticle evaluated by ^{1}H NMR

mPEG-*b*-(PCDM1-*co*-PCDD), mPEG-*b*-(PCDM1-*co*-PCMP), and mPEG-*b*-(PCDM1-*co*-PCMEE) were dissolved in deuterated acetonitrile (CD_3CN) at a concentration of 2 mg/mL (0.5 mL each). 0.3 mL of each solution was added to 0.7 mL of stirring D_2O to form polycarbonate-DM1 conjugate nanoparticles. The resulting polymer assembly solutions were analyzed using ^{1}H NMR spectroscopy (800 MHz), with D_2O selected to lock the field. Some characteristic peaks exhibited slight shifts in ^{1}H NMR due to the mixed solution.

1.7 Determination of critical micelle concentrations (CMCs) for mPEG-b-(PCDM1-co-PCDD), mPEG-b-(PCDM1-co-PCMP), and mPEG-b-(PCDM1-co-PCMEE).

The critical micelle concentration (CMC) of amphiphilic polycarbonate-DM1 conjugates in nanopure water was determined using pyrene as a fluorescent probe. Stock solutions of pyrene in acetone (6 µg/mL) and freshly prepared mPEG-*b*-(PCDM1-*co*-PCDD), mPEG-*b*-(PCDM1-*co*-PCMP), and mPEG-*b*-(PCDM1-*co*-PCMEE) (each at 0.2 mg/mL) were prepared. 100 µL of the pyrene stock solution was added to glass vials, which were left open at room temperature

for 30 minutes to allow the organic solvent to evaporate. Subsequently, 5 mL of polycarbonate-DM1 nanoparticle solutions at varying concentrations (ranging from 0.1 to 0.0004 mg) were added to the pyrene-containing vials, achieving a final pyrene concentration of 0.12 μg/mL in each vial. A control group containing pyrene in water was also prepared.

All sample solutions were stirred at 40 °C for 2 hours to facilitate the incorporation of pyrene into the micelles, and then allowed to equilibrate at room temperature for an additional 18 hours,. Fluorescence measurements were conducted at room temperature, with pyrene excited at 335 nm and its emission spectrum recorded from 350 to 500 nm. Both excitation and emission bandwidths were set to 3 nm. The intensity ratios at 372 nm and 383 nm, corresponding to the first and third vibrational peaks of pyrene, respectively, were plotted against the polymer concentrations in the sample solutions. The CMC was determined by fitting the intensity ratio against the polymer concentrations.

1.8 Long-term stability of mPEG-b-(PCDM1-co-PCDD)-NPs, mPEG-b-(PCDM1-co-PCMP)-NPs, and mPEG-b-(PCDM1-co-PCMEE)-NPs

The long-term stabilities of mPEG-*b*-(PCDM1-*co*-PCDD)-NPs, mPEG-*b*-(PCDM1-*co*-PCMP)-NPs, and mPEG-*b*-(PCDM1-*co*-PCMEE)-NPs were assessed by measuring size changes at different time points under various storage conditions using dynamic light scattering (DLS). Aqueous solutions of mPEG-*b*-(PCDM1-*co*-PCDD)-NPs, mPEG-*b*-(PCDM1-*co*-PCMP)-NPs, and mPEG-*b*-(PCDM1-*co*-PCMEE)-NPs (each at 100 μg/mL) were stored at room temperature and at 4 °C. After predetermined time intervals (ranging from 0 to 30 days), the samples were analyzed by DLS to record changes in size and size distribution.

1.9 DM1 release from mPEG-b-(PCDM1-co-PCDD)-NPs, mPEG-b-(PCDM1-co-PCMP)-NPs, and mPEG-b-(PCDM1-co-PCMEE)-NPs

The release of DM1 from mPEG-*b*-(PCDM1-*co*-PCDD)-NPs, mPEG-*b*-(PCDM1-*co*-PCMP)-NPs, and mPEG-*b*-(PCDM1-*co*-PCMEE)-NPs under different GSH concentrations was determined using high-performance liquid chromatography (HPLC). Buffered solutions with varying GSH concentrations (0, 50 nM, and 5 mM) were prepared by dissolving GSH in phosphate-buffered saline (PBS) and adjusting the pH to 7.4. A total of 200 μL of stock solutions of mPEG-*b*-(PCDM1-*co*-PCDD)-NPs, mPEG-*b*-(PCDM1-*co*-PCMP)-NPs, and

mPEG-*b*-(PCDM1-*co*-PCMEE)-NPs (each at 100 μg/mL) were thoroughly mixed with 800 μL of the GSH buffered solution, resulting in final working concentrations of 20 μg/mL. The released DM1 was monitored *via* HPLC at various time points. An Avantor C8 column was employed, using a gradient acetonitrile-H_2O eluent condition (5% CH_3CN for the first 2 minutes, followed by a gradient increase to 95% CH_3CN over 10 minutes) at a flow rate of 2 mL/min for detection. The compound eluted at 13.7 minutes and was detected by the UV detector at 230 nm.

1.10 Cell cultures

Hela cells, MDA-MB-231, and hTERT-HPNE cells were incubated in Dulbecco's Modified Eagle's Medium (DMEM) containing 10% of fetal bovine serum (FBS) and 1% of penicillin/streptomycin. PC-3 cells were incubated in F12K medium containing 10% of FBS and 1% of penicillin/streptomycin. Cells were maintained in a humidified cell culture incubator at 37 °C and 5% CO_2. For subculture, the cells were harvested from the cell culture medium by incubating in a trypsin solution for 3 min. After centrifugation for 3 min at 5000 rpm, the supernatant was discarded, and the fresh medium was added to neutralize the residual trypsin. The cells were resuspended in the fresh serum-supplemented medium and cultured in the humidified incubator at 37 °C and 5% CO_2.

1.11 Preparation of Nile red encapsulated polycarbonate-DM1 NPs

The hydrophobic fluorescent dye Nile red was encapsulated in all mPEG-*b*-(PCDM1-*co*-PCDD)-NPs, mPEG-*b*-(PCDM1-*co*-PCMP)-NPs, and mPEG-*b*-(PCDM1-*co*-PCMEE)-NPs for micellar labeling. In detail, the mixed solution in THF of Nile red (32 μg/mL) and different polymers, mPEG-*b*-(PCDM1-*co*-PCDD), mPEG-*b*-(PCDM1-*co*-PCMP), and mPEG-*b*-(PCDM1-*co*-PCMEE) (1 mg/mL each), were prepared, respectively. 200 μL of the above mixed solutions were added dropwise into 2 mL of water under stirring at room temperature, and the mixture continued to stir for 2 hours. To purify the micelles, the solutions were subjected to a continuous air stream over the surface to facilitate the evaporation and removal of the volatile THF. The solution volumes were then quantified to 2 mL by adding water to ensure a final polymer concentration of 100 μg/mL. Finally, the solutions were filtered using syringe filters to remove any undissolved free Nile red. The micelle sizes, excitation and emission spectra were then measured with DLS and fluorometer.

1.12 Uptake of polycarbonate-DM1 NPs into the cells

PC-3 cells were seeded in glass bottom dishes at a density of 100,000 cell per dish. After 24 hours to allow cell attachment, the cells were respectively treated with mPEG-*b*-(PCDM1-*co*-PCDD)-NPs, mPEG-*b*-(PCDM1-*co*-PCMP)-NPs, and mPEG-*b*-(PCDM1-*co*-PCMEE)-NPs, each at a DM1 concentration of 5 μM. The cells were incubated at 37 °C and 5% CO_2 for 1.5 h before the medium was removed. After washing three times with PBS, the cells were fixed with a formaldehyde solution (4% w/v in PBS) for 15 min at room temperature. The fixative solution was then removed, and the cells were washed with PBS. A Hoechst 33342 solution (1 μg/mL) was added to stain the cell nuclei. After removing the dye solution and washing with PBS, the cell samples were mounted for confocal fluorescent imaging. The Nile red labelled micelles were detected in the red channel (excitation 561nm, emission 570-651 nm), and the nucleus were detected in the blue channel (excitation 405 nm, emission 410-481 nm).

1.13 Evaluation of cytotoxicity of nanoparticles with MTT assay

The cytotoxicity of DM1, mPEG-*b*-(PCDM1-*co*-PCDD)-NPs, mPEG-*b*-(PCDM1-*co*-PCMP)-NPs, and mPEG-*b*-(PCDM1-*co*-PCMEE)-NPs cells were evaluated using MTT assays. The cells were seeded at a density of 3,000 cells per well and incubated for 24 h for attachment in 96-well plate. Then the medium was aspirated, and cells were treated with fresh medium containing different concentrations of of DM1, mPEG-*b*-(PCDM1-*co*-PCDD)-NPs, mPEG-*b*-(PCDM1-*co*-PCMP)-NPs, and mPEG-*b*-(PCDM1-*co*-PCMEE)-NPs, respectively, and incubated for 72 h. 3-(4,5-Dimethylthazol-2-yl)-2,5-diphenyltetrazolium bromide (MTT) solution (20 μL, 5 mg/mL) were added into each well and continued incubation for additional 4 h for the conversion of MTT to formazan by cellular reductase enzymes. After the medium was aspirated carefully, the cells were lysed using 100 μL of DMSO and homogenized with gentle shake at room temperature. The absorption at 570 nm of each well was measured using the plate reader. Untreated cells were used as a control and the relative cell viability was calculated and analyzed using GraphPad Prism 8.

2. Supplemental Figures and Tables

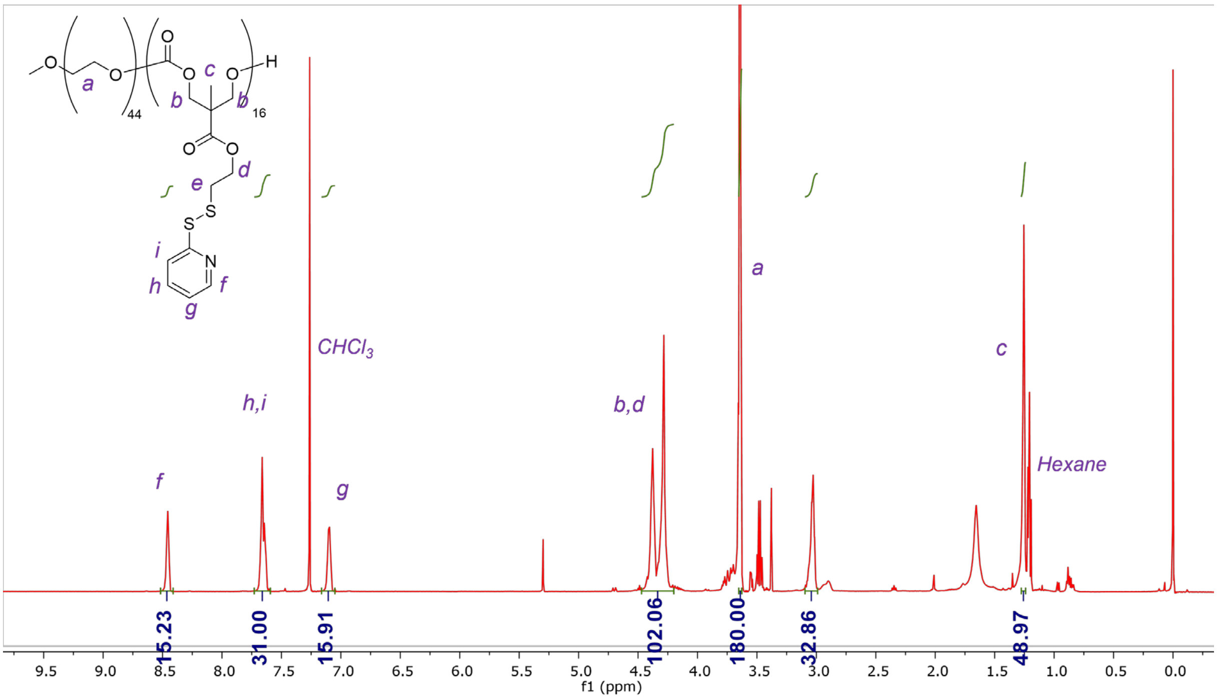

Figure S1. ^{1}H NMR spectrum of mPEG-*b*-PCPy in CDCl3

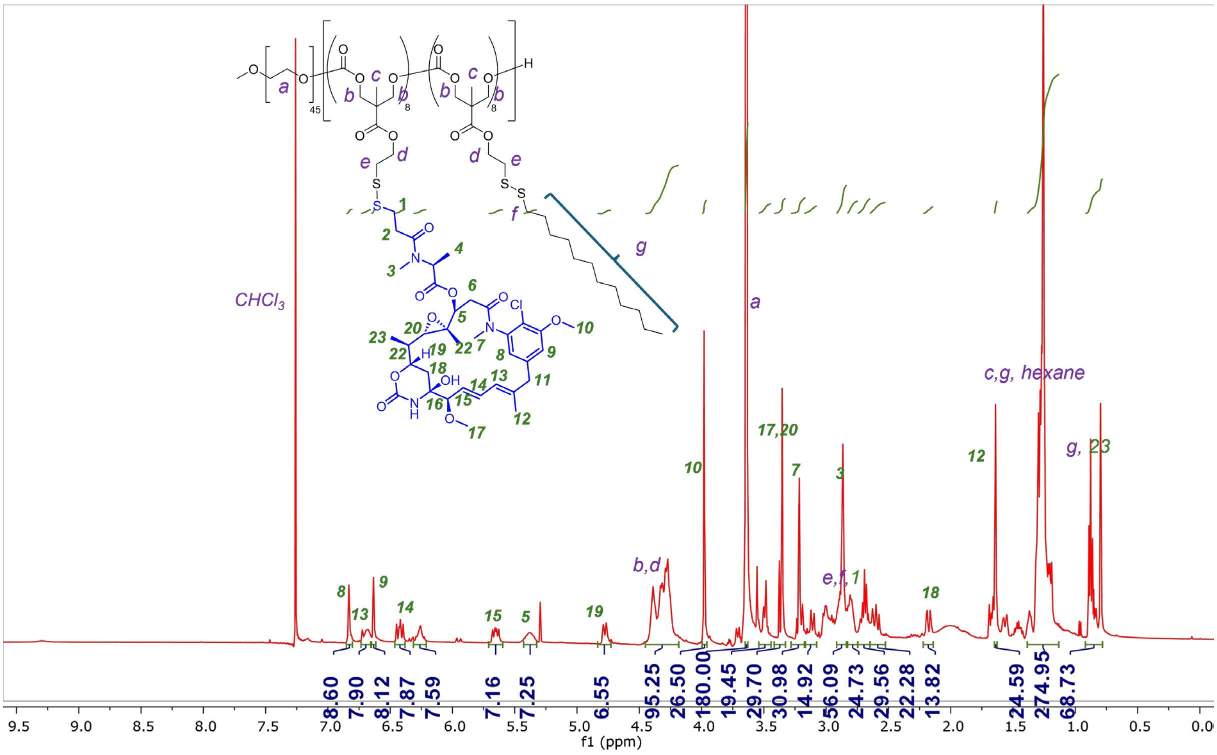

Figure S2. ^{1}H NMR spectrum of mPEG-*b*-(PCDM1-*co*-PCDD) in CDCl3

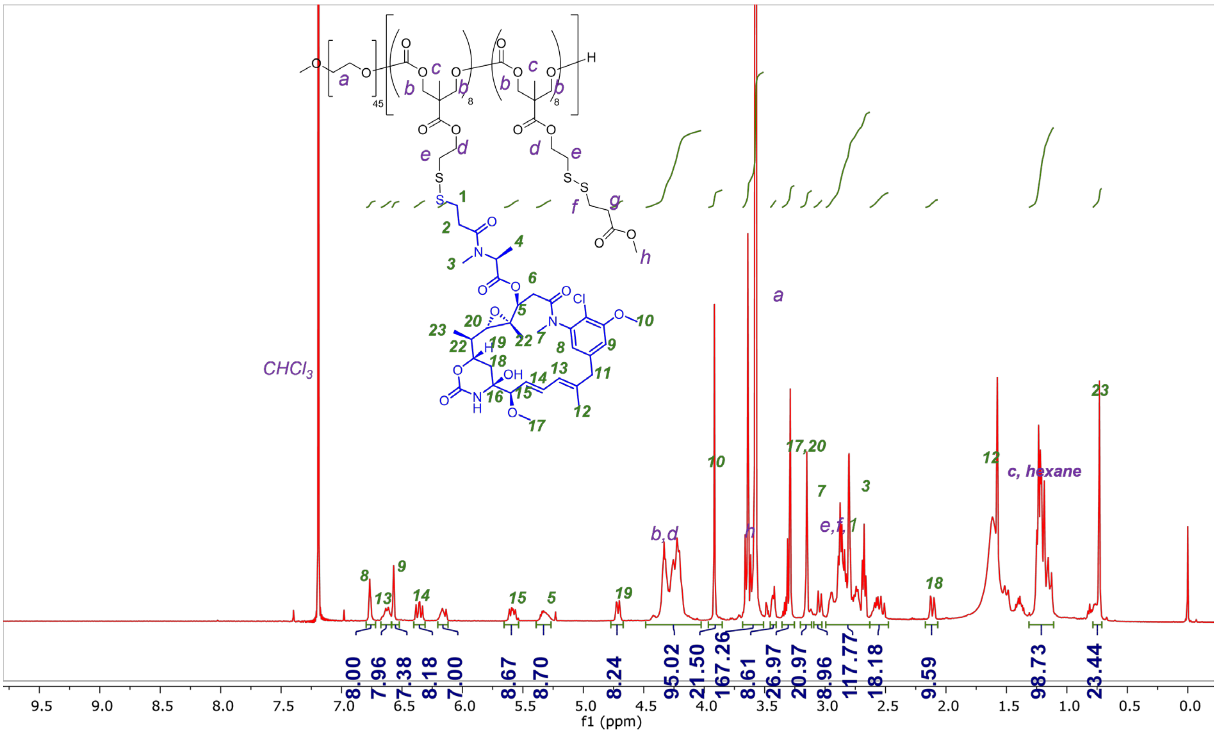

Figure S3. ^{1}H NMR spectrum of mPEG-*b*-(PCDM1-*co*-PCMP) in $CDCl_3$

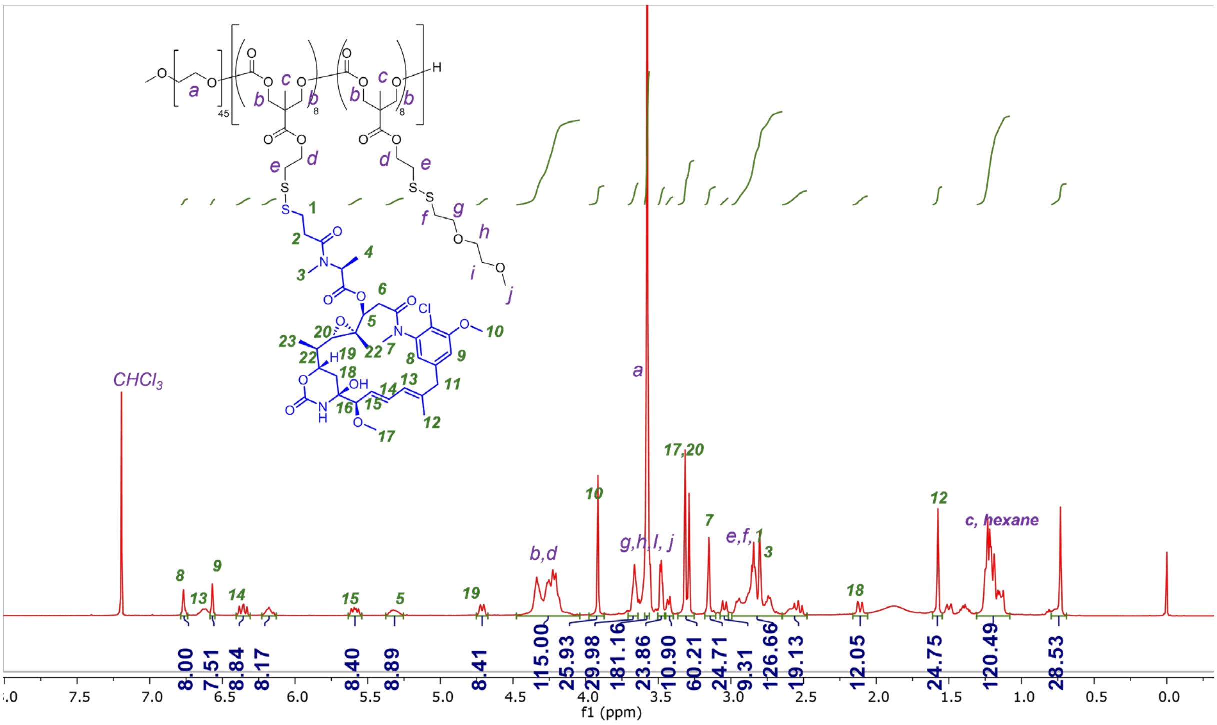

Figure S4. ^{1}H NMR spectrum of mPEG-*b*-(PCDM1-*co*-PCMEE)

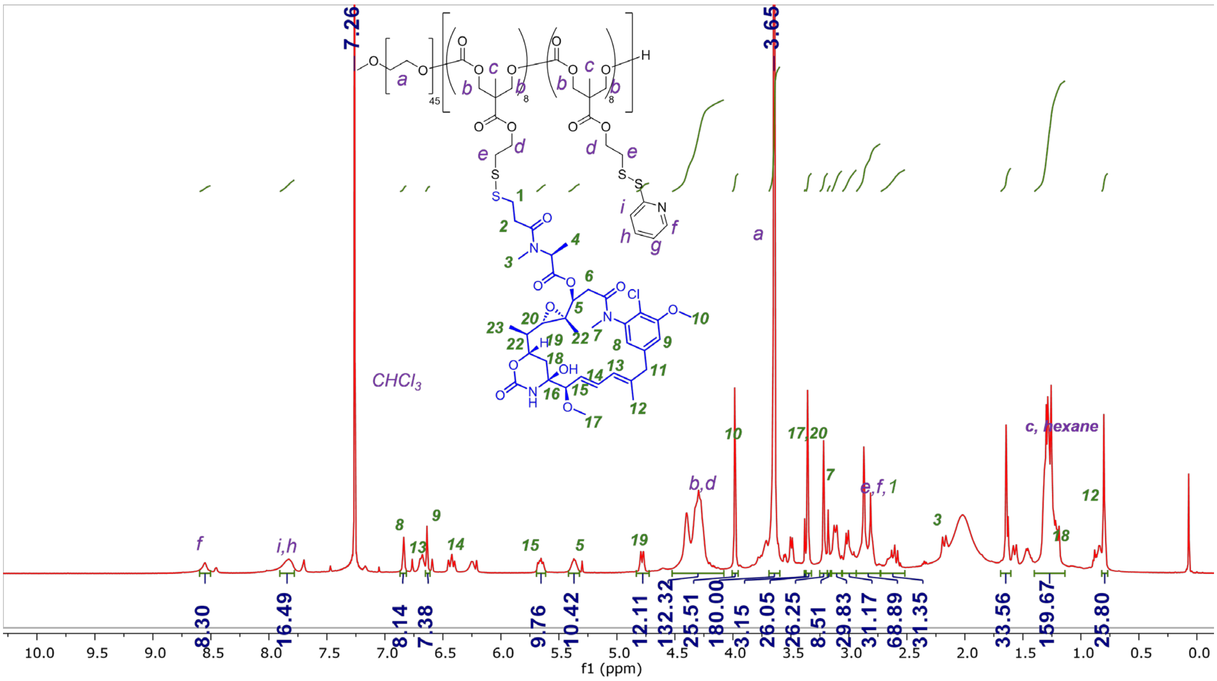

Figure S5. ^{1}H NMR spectrum of mPEG-*b*-(PCDM1-*co*-PCPy) in $CDCl_3$

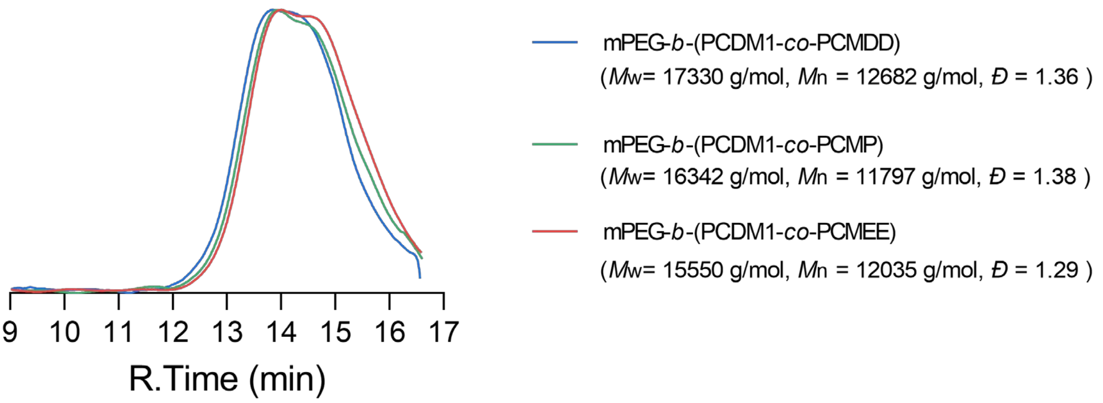

Figure S6. Size exclusion chromatography traces of mPEG-*b*-(PCDM1-*co*-PCDD), mPEG-*b*-(PCDM1-*co*-PCMP), mPEG-*b*-(PCDM1-*co*-PCMEE)
